# How to Identify e-Cigarette Brands Available in the United States During 2020-2022: Development and Usability Study

**DOI:** 10.2196/47570

**Published:** 2024-02-28

**Authors:** Shaoying Ma, Aadeeba Kaareen, Hojin Park, Yanyun He, Shuning Jiang, Zefeng Qiu, Zidian Xie, Dongmei Li, Jian Chen, Richard J O’Connor, Geoffrey T Fong, Ce Shang

**Affiliations:** 1 Center for Tobacco Research The Ohio State University Wexner Medical Center Columbus, OH United States; 2 Department of Computer Science and Engineering The Ohio State University Columbus, OH United States; 3 Department of Clinical and Translational Research University of Rochester Medical Center Rochester, NY United States; 4 Department of Health Behavior Roswell Park Comprehensive Cancer Center Buffalo, NY United States; 5 Department of Psychology University of Waterloo Waterloo, ON Canada; 6 School of Public Health Sciences University of Waterloo Waterloo, ON Canada; 7 Ontario Institute for Cancer Research Toronto, ON Canada; 8 Department of Internal Medicine Division of Medical Oncology The Ohio State University Wexner Medical Center Columbus, OH United States

**Keywords:** tobacco, electronic cigarette, e-cigarette, electronic nicotine delivery systems, electronic nicotine delivery system, vaping, market surveillance, tobacco marketing

## Abstract

**Background:**

Prior studies have demonstrated that the e-cigarette market contains a large number of brands. Identifying these existing e-cigarette brands is a key element of market surveillance, which will further assist in policy making and compliance checks.

**Objective:**

To facilitate the surveillance of the diverse product landscape in the e-cigarette market, we constructed a semantic database of e-cigarette brands that have appeared in the US market as of 2020-2022.

**Methods:**

In order to build the brand database, we searched and compiled e-cigarette brands from a comprehensive list of retail channels and sources, including (1) e-liquid and disposable brands sold in web-based stores, (2) e-cigarette brands sold in brick-and-mortar stores and collected by the Nielsen Retail Scanner Data, (3) e-cigarette brands compiled by Wikipedia, (4) self-reported e-cigarette brands from the 2020 International Tobacco Control Four-Country Smoking and Vaping (ITC 4CV) US survey, and (5) e-cigarette brands on Twitter. We also estimated the top 5 e-cigarette brands by sales volume in brick-and-mortar stores, by the frequency and variety of offerings in web-based shops, and by the frequency of self-reported brands from the 2020 ITC 4CV US survey.

**Results:**

As of 2020-2022, a total of 912 e-cigarette brands have been sold by various retail channels. During 2020-2022, the top 5 brands are JUUL, vuse, njoy, blu, and logic in brick-and-mortar stores; blu, king, monster, twist, and air factory for e-liquids in web-based stores; hyde, pod mesh, suorin, vaporlax, and xtra for disposables sold in web-based stores; and smok, aspire, vaporesso, innokin, and eleaf based on self-reported survey data.

**Conclusions:**

As the US Food and Drug Administration enforces the premarket tobacco market authorization, many e-cigarette brands may become illegal in the US market. In this context, how e-cigarette brands evolve and consolidate in different retail channels will be critical for understanding the regulatory impacts on product availability. Our semantic database of e-cigarette brands can serve as a useful tool to monitor product and marketplace development, conduct compliance checks, assess manufacturers’ marketing behaviors, and identify regulatory impacts.

## Introduction

Electronic cigarettes or e-cigarettes, were introduced in the US market in 2007 [1]. As of 2022, a total of 2.55 million US high school (14.1%) and middle school (3.3%) students reported using e-cigarettes in the past 30 days [2]. Unlike cigarettes, the e-cigarette market is highly diverse, with a wide range of product configurations (eg, rechargeables vs disposables) and features (eg, flavors and nicotine concentrations). e-Cigarette market has also been characterized as highly dynamic, with leading brands constantly changing [3-7]. As of 2018, the e-cigarette market was valued at US $3.5 billion, with a large number of brands (>460) and flavors (>7700) [8,9].

The number of e-cigarette brands is an important indicator of market concentration and competitiveness, which measures the ability of a single firm, or a small group of firms, to exert monopoly power [10,11]. For example, the cigarette market is characterized as a highly concentrated oligopolistic cigarette market, with a few large cigarette companies accounting for the majority of market shares [10,12]. In contrast, before JUUL uptake, the US e-cigarette market was very competitive and comprised hundreds, if not thousands, of small manufacturers selling products to consumers and retailers [13]. However, in recent years, cigarette companies have increasingly owned or invested in e-cigarette products, taking over a significant share of the e-cigarette market [14]. As a result, the e-cigarette market may have become less competitive over time and the cigarette and e-cigarette markets are growing integrated [15].

Constructing a database of e-cigarette brands is crucial to monitor market concentration, competitiveness, and development, especially for the US e-cigarette marketplace. However, such a database has not been established yet. As prior studies demonstrated, e-cigarette retails are dispersed in various retail channels, including brick-and-mortar stores, web-based stores, and vape shops or specialty stores [16,17]. However, only products and brands sold in brick-and-mortar stores are well-tracked by the Nielsen Retail Scanner Data. Little is known about e-cigarette brands and products sold in vape shops and web-based stores. Moreover, the few studies that examine products sold in these alternative channels suggest that products found in vape shops and web-based stores are significantly different from those in brick-and-mortar stores in terms of prices and product characteristics [18,19]. Therefore, a comprehensive database of brands is needed to identify brand differences by retail channels and assess accurately the e-cigarette market concentration and competitiveness.

A database of e-cigarette brands can also be used to identify regulatory impact by tracking the life cycle of a product and conducting compliance checks by identifying illegally sold products. In response to the substantial rise in e-cigarette use among US youth, the regulatory environment has evolved with many policy changes or proposals in recent years [20,21]. The available brands and types of e-cigarette products in the US market have been affected by the federal-level regulatory actions taken by the Food and Drug Administration (FDA) as well as state- and local-level policies that aim to curb e-cigarette use [3,21,22]. It is therefore highly warranted to have a live database of brands sold in the marketplace, which can demonstrate whether products with prohibited attributes are dropped from the market and how brands are evolving to comply with regulations.

Another function of a brand database is to assist in compliance checks. As the US FDA enforces the premarket tobacco market authorization (PMTA), many e-cigarette brands may become illegal in the US market [23]. A database of e-cigarette brands will allow the FDA and state and local agencies to identify if a certain brand is illegal or has been approved by the FDA. The brand database can also inform how e-cigarette brands evolve and consolidate with the enforcement of PMTA and answer the question of whether the PMTA makes the e-cigarette market less competitive in terms of limiting the availability of products [24,25].

The US marketplace of e-cigarette products is dynamic and rapidly changing. Therefore, a database of e-cigarette brands is also critical to tracking social media brand mentions and identifying emerging brands [26-29]. There have been attempts to identify mentions of e-cigarettes’ brand names and flavors on social media, such as Twitter (Twitter, Inc) [5,30], Reddit (Reddit Inc) [30], YouTube (Google) [29], and Instagram (Meta Platforms) [31], and to identify and classify emerging flavors in the web-based market [32], using machine learning algorithms. However, the lack of a comprehensive semantic database of e-cigarette brands that capture different purchasing channels has hindered the efforts of using these language-based algorithms to efficiently identify brands.

Given the importance of an e-cigarette brand database, this study aims to construct such a database and further assess popular and common brands reported from different sources. The database also provides a snapshot of the products that have appeared in the market as of 2022, allowing for future studies that analyze marketplace development and policy impacts.

## Methods

### Data Sources

We searched comprehensively and identified 6 sources that we used to create the semantic database of e-cigarette brands. These include (1) existing brand websites surveillance [8] that reported on a pre-determined list of e-cigarette brands; (2) e-cigarette brands sold in the brick-and-mortar stores reported by the Nielsen Retail Scanner Data, accessed through the Kilts Center for Marketing at the University of Chicago Booth School of Business [33]; (3) Wikipedia’s “list of electronic cigarette and e-cigarette liquid brands,” which was established by a community of volunteers who add brand names from data sources including peer-reviewed journal articles, news articles, reports from antitobacco organizations, and other sources, and we accessed it on November 29, 2022 [34]; (4) a list of e-cigarette brands mentioned on Twitter from May 2021 to December 2021 [5]; (5) self-reported brands from the 2020 International Tobacco Control Policy Evaluation Project’s Four-Country Smoking and Vaping (ITC 4CV) US survey; and (6) e-cigarette brands collected using web scraping of 5 popular web-based stores. The scraping data captured a wide range of e-liquid and disposable e-cigarette products and brands sold on the web, which were traditionally not well captured by other sources. The scraping data contained information of over 16,000 unique e-liquid or disposable products collected during 2021-2022. Additional details are available in Multimedia Appendix 1.

### Brand Identification

For web-based store brands, the same brand could be written in slightly different ways across stores, and we observed variations caused by (1) spaces or hyphens (such as “sad boy” vs “sadboy”); (2) pluralization (such as “bad drip” vs “bad drips”); (3) suffixes (such as “barista brew” vs “barista brew co.,” and “mr. good” vs “mr. good vape”); (4) abbreviations or aliases (such as “naked 100” vs “NKD 100”); and (5) misspelling (such as “coastal clouds” vs “costal clouds”). Using algorithms, we identified 237 unique e-liquid brand 97 unique disposable e-cigarettes in 2021-2022.

We used STATA/SE (version 17.1; StataCorp) software to convert all brand names to lowercase and remove duplicates. For brands with more than 1 product line, a research specialist from our team looked up relevant information on the web and identified them so that we extracted each brand name with multiple product lines and collections and further cleaned up the brand database. A total of 4 members from our research team reviewed the brand list to ensure accuracy. We then used this list of e-cigarette brands (based on Nielsen, Wikipedia, and data collected from web-based stores), to identify and match with the self-reported e-cigarette brands in ITC 4CV Wave 3 (2020) survey data.

Among 1696 observations from the self-reported brand variables in the ITC survey, 181 (10.7%) of them contained irrelevant or insufficient information, such as “don't know,” and “its unbranded from the market.” For the remaining 1515 observations, 720 (47.5%) were successfully identified and matched using our initial brand list from 3 sources (Nielsen, Wikipedia, and our unique e-liquid data scraped from web-based stores). Aided by algorithms, we checked the rest of the self-reported brands that were unmatched or unidentified both by humans and by machines and extracted brand information from those observations. Since the same brand can be reported in different ways such as with or without space, we consolidated the brand names by removing the spaces and documented possible variations of a unique brand, such as “geekvape” and “geek vape” in Multimedia Appendix 2. Consequently, 167 new brand names from the ITC 4CV survey were added to our brand database.

Based on our disposable e-cigarette brand data collected from web-based stores in 2022 and the list of e-cigarette brands on Twitter from May 2021 to December 2021 collected by Tang et al [5], we then updated our brand database and in total 138 new e-cigarette brands were added from those 2 data sources.

### Identifying Top Brands

For products sold in brick-and-mortar stores, we used the Nielsen Retail Scanner data to identify the top 5 brands with the greatest sales in 2020. For self-reported brands, we used the frequency counts in the 2020 ITC 4CV US survey to identify the top brands. For products sold in web-based stores, we use the frequencies of offering (number of unique products of a brand multiplied by the number of stores that offer the brand) to identify the top 5 brands for e-liquid and disposable products, respectively.

### Ethical Considerations

In this study, we compiled data on e-cigarette brands using sources including peer-reviewed publications about brand surveillance and brand mentions [5,8], the Nielsen Retail Scanner Data [33], Wikipedia [34], a tobacco use survey, and our unique data scraped from e-cigarette web-based stores. Thus, no human subjects were involved, and the determination of no human subjects was approved by the Ohio State University institutional review board. The survey protocols and all materials including the survey questionnaire for the 2020 (Wave 3) ITC 4CV US survey were cleared for ethics by the Research Ethics Board, University of Waterloo, Canada (REB#20803/30570) and the Medical University of South Carolina (waived due to minimal risk).

## Results

In total, we identified 912 e-cigarette brands available in the United States during 2020-2021 and presented this database in Multimedia Appendix 2. As we compiled the brand database from multiple sources, we observed that e-cigarette manufacturers are creative when naming their product brands; in some cases, generic terms such as “z,” “e s,” “pods,” “something,” “mix,” and “e-hookah” are used as brand names. There are also brand names like “zoom” that could lead to ambiguous search terms and false results. We suggest that for those brands, researchers could use the brand names along with search terms such as “vape” as search terms to conduct market surveillance (eg, social media monitoring) and avoid false results.

The top 5 brands from different retail channels and resources are presented in Textbox 1, which presents the top 5 brands (by sales volume in counts) in the Nielsen Retail Scanner Data during 2020, the top 5 e-liquid brands (by frequency counts) in the scraped data during 2021, the top 5 disposable e-cigarette brands (by frequency counts) in the scraped data during 2022, and the top 5 self-reported brands (by frequency counts) in the ITC 4CV US survey during 2020.

**Top e-cigarette brands available in the United States during 2020-2022, based on different data sources.**

Nielsen Retail Scanner Data, 2020 (top 5 brands by sales volume in counts): JUUL, vuse, njoy, blu, and logic

e-Liquid data from popular web-based vape shops, 2021 (top 5 brands by frequency counts): blu, king, monster, twist, and air factory

Disposable e-cigarette data from popular web-based vape shops, 2022 (top 5 brands by frequency counts): hyde, pod mesh, suorin air bar, vaporlax, and xtra

International Tobacco Control Four-Country Smoking and Vaping (ITC 4CV) US Survey data, 2020 (top 5 brands by frequency counts): smok, aspire, vaporesso, innokin, and eleaf

Focused on the sales of e-cigarette products from brick-and-mortar stores, Nielsen Retail Scanner Data captured 82 brand or model names (567 product Universal Product Codes [UPCs]) in the “ELECTRONIC CIGARETTES–SMOKING” product module in 2020. Of the 82 brands or models, 59 sold at least 2 unique e-cigarette products. The top 5 high-level e-cigarette brand names by the number of products (ie, UPCs) in Nielsen Retail Scanner Data were njoy (70 product UPCs), vuse (64 product UPCs), blu (63 product UPCs), logic (31 product UPCs), and JUUL (30 product UPCs), which together accounted for about 45.5% (n=258) of total e-cigarette products observed in the Nielsen data. Product types varied, such as e-liquids, replacement pods, nonreplacement pods, prefilled cartridges or tanks, disposables, devices (eg, pod and mod), and starter kits (both device and pods or cartridges). Furthermore, during 2020 in brick-and-mortar stores, the top 5 brands (in descending order) by sales volume in counts were JUUL, vuse, njoy, blu, and logic, which represented about 137 million (97.9%) out of 140 million total e-cigarette sales volume in Nielsen Retail Scanner Data.

Based on the web-based store data, the top 5 e-liquid brands by frequency counts (reported in parentheses) were blu (780), king (553), monster (531), twist (488), and air factory (480). Here the frequency count for each top e-liquid brand was calculated as the number of different products from this brand offered by 5 stores, that is, a number of stores offering multiplied by brand variations. The top 5 disposable e-cigarette brands by frequency counts (reported in parentheses) sold by 5 web-based vape shops were hyde (214), pod mesh (126), suorin air bar (125), vaporlax (124), and xtra (115), which were different from the top brands in Nielsen data. Interestingly, self-reported data suggested top brands that differ from both web-based stores and Nielsen data. Based on the ITC 4CV Wave 3 US survey data, during 2020, the top 5 brands by frequency counts (ie, how often were they mentioned by participants, reported in parentheses) were smok (201), aspire (104), vaporesso (76), innokin (65), and eleaf (64).

## Discussion

Prior research used keyword searches and identified over 460 e-cigarette brands through January 2014, but the market has grown exponentially since then [35]. Although existing evidence shows that the number of e-cigarette brands has not increased much since 2014, this conclusion relied on limited resources or retail channels [8]. In this study, we used 6 different data sources to consolidate brands identified from multiple retail channels (brick-and-mortar stores, web-based stores, social media mentions, and self-reported data) and identified 912 unique e-cigarette brands as of 2020-2022. This suggests that a large number of e-cigarette brands existed in the market before the enforcement of PMTA, which is in line with the existing assessment that the e-cigarette market is more competitive than cigarettes with many e-cigarette manufacturers [10,13]. However, as tobacco companies increase their shares in the e-cigarette market by producing their own e-cigarette brands or purchasing existing e-cigarette brands, future research could use our database to map brands with tobacco company ownerships to better understand tobacco companies’ interest in e-cigarettes and the changes in the competitiveness and concentration of the e-cigarette market [15].

Regulation on e-cigarettes such as the FDA’s PMTA action might reduce competition in the marketplace, as e-cigarette brands owned by big tobacco companies may have a greater capacity to properly respond to regulatory actions (eg, file a PMTA) and remain in the market [24]. In addition, recent evidence suggests that in response to the FDA regulations that ban flavors other than menthol and tobacco in prefilled cartridges, the marketplace and consumers switch to disposables that provide a spectrum of flavors [3]. Therefore, it is important to track market development, product availability, manufacturers’ market power (ie, market concentration or competitiveness), and market responses to regulations, which this brand database can help.

Since 2021, the FDA has issued marketing denial orders (MDOs) to a number of e-cigarette companies, including prohibiting sales and distribution of all products from JUUL including devices and pods [21,36]. The FDA maintains an updated list of companies (instead of brand names) that have products currently marketed in the United States and have been issued MDOs [36]. It also announces the latest marketing decisions about e-cigarette companies and specific e-cigarette brands and models that have received MDOs [36]. After comparing the brands in the FDA’s marketing decisions from October 12, 2021, to January 24, 2023 [36], with our brand database, we found that all brands in those decisions were included in our database, which supports the validity and completeness of our database, which goes beyond the FDA list with additional brands that are not included in the decision list. This comparison also supports the potential of using this brand database to conduct compliance checks and monitor market product availability that could aid policy making.

Existing evidence from other studies shows that e-cigarette brands owned by tobacco companies typically offer a limited range of e-cigarette products, while brands owned by vape shops are much more likely to have a diverse range of flavor and nicotine options [8]; e-cigarette brands and product types are among the most important factors that influence consumers’ purchasing choices [8,37]. We also show that the most frequently mentioned e-cigarette brands by participants in a tobacco survey, the top brands by sales volume in brick-and-mortar stores based on Nielsen Retail Scanner data, and the top brands by product availability in web-based vape shops are very different, which demonstrates the importance of obtaining information from multiple data sources and purchasing channels when calculating the market share of various e-cigarette brands and the market concentration and power of each brand in the quickly changing e-cigarette marketplace.

We report the top 5 disposable e-cigarette brands (by frequency counts) from our 2022 scraped data as well as the top 5 e-liquid brands (by frequency counts) from our 2021 scraped data. Our database includes e-cigarette brands that are predominately sold in web-based stores; in particular, e-liquid brands are typically not well captured in existing data sources (eg, Nielsen Retail Scanner Data) and our database makes a contribution to the literature by providing those brands. For e-cigarette brands sold on the web, we have also observed that web-based vape shops sell a variety of products with different flavors, nicotine levels, and forms, suggesting the appeals of these brands sold in web-based stores [18,19,32,38,39].

Furthermore, to the best of our knowledge, our database containing brand names from 6 different data sources is the most comprehensive and up-to-date, and could contribute to tobacco regulatory science in multiple ways. In addition to compliance checks, product availability assessments, and market power estimations, the brand database can be used to conduct social media market surveillance using natural language processing and other machine learning techniques [40,41]. Specifically, brand search terms are key to identifying whether e-cigarettes are being mentioned in social media posts or on a website [5,31,42,43]. Therefore, our database not only provides a comprehensive list of e-cigarette brand search terms but also allows for the identification of emerging brands through techniques such as name entity recognition. Future research may also expand this database by linking e-cigarette brands with product characteristics such as flavors, nicotine levels and forms, and device types (disposables vs cartridges vs e-liquid; open vs closed systems) to facilitate rapid market surveillance.

Another potential function of this database is to assist in future tobacco survey development by allowing for a dropdown list that reflects the complexity of the e-cigarette marketplace and brands [2,44-48]. Our experience with the ITC 4CV survey suggests that respondents may have difficulties in self-reporting e-cigarette brands in an open-ended question. A drop-down list could enhance the ability of surveys to capture brands accurately.

Our study has some caveats. The brand names from our web-based vape shop data reflect a snapshot of e-liquid brands sold in popular web-based stores in 2021. As we continue our web scraping efforts, we will be able to extract brand information for a wide range of e-cigarette products sold in the web-based market, such as disposables, devices, and starter kits. Nonetheless, the e-liquid and disposable e-cigarette brand data from web-based vape shops and the list of e-cigarette brands on Twitter complements the brand information in brick-and-mortar stores from the Nielsen data, the existing list of brand names from Wikipedia (accessed on November 29, 2022), and self-reported brands from survey participants during 2020, together making our brand database the most comprehensive and up-to-date to the best of our knowledge. Another limitation is that brands in specialty vape shops are not necessarily captured in our semantic database. Future research is warranted to assess the brand information from specialty vape shops by paying visits to the stores and conducting qualitative interviews with store owners and staff members.

In conclusion, the development of a comprehensive semantic database that contains e-cigarette brands in the US during 2020-2022 demonstrates the competition of the existing e-cigarette market, as well as the use of novel techniques such as name entity recognition to identify emerging brands. It also has broader public health implications on the need to continuously monitor market concentration and manufacturers’ responses to regulations. Our database can be used to facilitate compliance checks and rapid surveillance of product availability to inform policy making.

## Appendix

Multimedia Appendix 1 Additional details of the methodology used to create the brand database.

Multimedia Appendix 2 E-cigarette brand names available in the United States during 2020-2022.

## Abbreviations

FDA: Food and Drug Administration
ITC 4CV: International Tobacco Control Four-Country Smoking and Vaping
MDO: marketing denial order
PMTA: premarket tobacco market authorization
UPC: Universal Product Code

## Data Availability

All data generated or analyzed during this study are included in this published article and Multimedia Appendices 1 and 2.
