# Supplementary material for: How to Identify e-Cigarette Brands Available in the United States During 2020-2022: Development and Usability Study
Source: JMIR Form Res. 2024 Feb 28;8:e47570. doi: 10.2196/47570 (PMC10938231; doi:10.2196/47570)
Supplement: Multimedia Appendix 1 [file formative_v8i1e47570_app1.pdf]

Multimedia Appendix 1

Additional details of the methodology used to create the brand database.

From the Nielsen Retail Scanner Data, we extracted brand information of products with “ELECTRONIC CIGARETTES – SMOKING” as the product module description. For each e-cigarette brand that appeared in the Nielsen Retail Scanner Data, sales volume in counts was calculated; the top five brands by sales volume are presented in *Table 1*.

Using web scraping algorithms[1,2], we extracted comprehensive product-level data on e-liquids in 2021 and on disposable e-cigarettes in 2022, including their brand information, from five US online vape shops. As detailed in Ma *et al*. (2022), we mask store identities in the analysis and refer to them as stores 1-5.[1]

Self-reported brands in the International Tobacco Control Four-Country Smoking and Vaping (ITC 4CV) survey were collected from participants’ answers to the following questions: the brand(s) of vaping device that the participant currently uses most often, the brand(s) of vaping device that the participant used last, the brand(s) of cartridges/pods they last purchased, and the brand(s) of the device they were using when the battery overheated, exploded, or caught on fire.

REFERENCES

1 Ma S, Jiang S, Ling M, *et al.* Excise taxes and pricing activities of e-liquid products sold in online vape shops. *Tob Control* Published Online First: 2022. doi:10.1136/tobaccocontrol-2021-057033

2 Ma S, Jiang S, Ling M, *et al.* Price Promotions of E-Liquid Products Sold in Online Stores. *Int J Environ Res Public Health* 2022;**19**. doi:10.3390/ijerph19148870
