# Supplementary material for: How to Identify e-Cigarette Brands Available in the United States During 2020-2022: Development and Usability Study
Source: JMIR Form Res. 2024 Feb 28;8:e47570. doi: 10.2196/47570 (PMC10938231; doi:10.2196/47570)
Supplement: Multimedia Appendix 2 [file formative_v8i1e47570_app2.pdf]

Multimedia Appendix 2

E-cigarette brand names available in the United States during 2020-2022.*
(based on six data sources detailed in the main text,
brand names all lowercase and separated by semicolon)

10 motives; 13th floor elevapors; 180 smoke; 180smoke; 21st century smoke; 4 aces; 4x pods; 528 custom vapes; 7 daze; 7's; 777 ecigs; 80v; 88 vape; 9 south vapes; a final course; aaok; advanced vapor devices; advken; air factory; airbar; airbender; airship nicsalt; aisu; aleader x-drip; all stars; alt; alt zero; alternacig; alternativ; americig; amigo itsuwa; anarchist; anml unleashed; antonio villard; apollo; aqua; aramax; arc; ariovape; arizer; armageddon; aromativ; artery; asmodus; aspire; atmos; atom 8; augvape druga 22 squonk; aurora; avail vapor; azure vaping; b+mor; backwoods; bad drip; bad drip labs; bam's cannoli; banana butt; bang; bantam; barista brew co.; basix; baton; beantown vapor; bear graham; beard; beard ryse; beatnic vapor; beetle; ben johnson's morning dew; benevolent; berryfornia; biasing; bidi vapor; big bar; big boy; billet box; bizzle; blac label; black magic; black note; black ops; blankzpods; blaze; blazz!; blitz; blnd'd; blng; blu; blvk; blvk unicorn; bmor; bo vaping; boho; bombies after dark; bora; boulder; breeze smoke; brella; broadstreet; buck naked; bud vape; bull smoke; bundle; burst; buzz; c & c apothecary; cabissi; cali greens; cali pods; california grown; candy king; carbon; caribbean cloud company; carnival; cassadaga; cbd fx; cbdfx; ccell; central vapors; cevo; chain vapez; charlie's chalk dust; chubby; ciberate; cig2o; cigalectric; cigartex; cigavette; ciggies; cigirex; cigr8; circus cookie; cirro; clayton; clean cig; clean cigs; clearette; cliq; cloud alchemist pod refills; cloud chasers inc.; cloud nurdz; cloud nurdz synthetic; cloudmouth; cloudv; cloudvapes; clown; clx; coastal clouds; coee; coilart; coilart mage; cola man; cones; confection; cosmic fog; cottien; council of vapor; country clouds; cream collection; crft; crisp; criss-cross; crumbz vapor; cube; cue; cuttwood; cuvana; cyclone pods; dank vape; dapr royal gambit; dat bar; davinci; deathwish; deep cuts; delicious pods; deliciously twisted flavors; demon vape; diamond mist eliquids; dicodes; digiflavor; dinner lady; direct eliquid; direct vapor; director's cut; dongguan wismec electronics co. ltd.; dotmod; dovpo; dr. crimmy; dr. salt; draco; dragonite international limited; drip bar; drip more; dripco; drop plus; dura true; durasmoke; dynavap; e s; e swisher; e tron; e-burn; e-hookah; e-lites; e-smokes; east indies hookah; ecblend flavors; ecigwizard; eclipse; ecloud; eco-cigs; ecopure; edge; ego; ehpro; ekaiser; eleaf; electric lotus; electric tobacconist; electronic cigarettes international group ltd; elektro; element; element e-liquids; element ns20; elf bar; elysian; enovap; envii fitt; envogue xl; eonsmoke; epic; epuffer; esco bars; ethos; etron 3t; ever smoke; eversmoke; evo; evolab; exotic bar; exxus; ez cig; ez smoker; fank; fantasia; faze; fifty-one; fiji fruits; fill my pod; fin; finest; finiti; firebrand; firefly; five pawns; fizzy; flavor vapes; flavored mist; flexvape; floss; flum; food fighter; forbo shisha 2 go; four seasons; free max; freemax; freeton; fresca; fresh & sour; fresh bar; fresh farm; fresh pressed; frozen vape co.; fruit monsta; fruitia plus; fryd; full chubs; fum; fumare; fumizers; fun drip; funkmaster; g-taste; gambler; gamucci; geek vape; geekvape; generic adult sours; geo; gilla; glamee; glas; glo; go2; gold flora; gold leaf; gorilla warfare; gost; grand vapor; grav labs; green puffer; green smart living; green smoke; green stix; greenlightvapes; greensmartliving; greensound; gulp; gummy o's; halcyon vapors; hale; halo; halo cigs; happy end; hato; haus; hcigar; hcigar vt inbox dna75; heartlandvapes; hellfire mods; hi-drip; high roller sweets; hippo; hitt; holy cannoli; hookah; hookah stix; horizontech; hot vapes; hqd; hsky vapor; humble; hyde; hyppe; hyve x chubby; i love salts; i smok; ice cream man; icon; ifrit; ijoy; ijoy capo squonk; ikhal; ikrusher; ileva; imecig; imperial; imperial hookah; imperial smoke; infamous; infzn; innevape; innokin; intellicig; invc; ipv; ipv technology co.; iqos mesh; ismok; ismoke vaper tech; ivape iq; j vapes; j'adore for her; jac vapour; jai; jak; jam monster; jasper jasper

capsol; jasper jasper impulse; jaybo; jazzy boba; jb vapor; jet; johnny be fresh; johnson creek; joost; jouz; joyetech; joymy; jubi; juice dimension; juice head; juice hog; juice house; juice man; juju royal; junkys stash; just juice; justfog; justpods; juul; kamry; kandypens; kanger; kanger lily; kangertech; kangvape; kayfun v4; keep it 100; kennedy vapor; khali; kik; kilo; kimsun; king; kingpen; kingtons; koi cbd; kraken; krave; kumiho; kush; kwit stick; lavapod; lawless; leaf buddi; leap; level up vapor; liberty flights; light-free; limelight; liqueen; liquid chronic; liquid nicotine wholesalers; liquid state; lite bar; lite-up anywhere; little dipper; loaded; logic; logic zero; lollidrip; longbeach original; loon; lost art; lost vape; lotus; lucid; lucky strike; lula vape; lulu; lunar rover; lung hit; lush; lyf; mad hatter; mad hatter juice i naked; mad vapes; magic mist; mamasan; marina; markten; marlboro heatsticks; matchless; mate 1; maxcore; medpharm; melting point extracts; mesh; met4; meta drop; metrix; mgo; mi-pod; micro brew vapor; midose; mig vapor; mighty vapor; milkman; millennium; minute man; mistic; mix; mlife; mngo; modefined; modefined lyra 200w; modern smoke; mods; mojo; mono; monster; moo; mosmo; moti; motley brew; mountain moon; mountain oak vapors; mouse chef; mpx; mr fog; mr. freeze; mr. good vape; mr. salt e; mr. salt-e; mr. vapor; mrkt; my myst; myblu; myle; myst labs; naked 100; nanostix; nasty; nbl-no company listed; ne where; nectar collector; neo; neo solo; nevoks; new smoke; nic5; nicmaxx; nicocig; nicolites; nicotek; nicotek llc-nbl; nicotek metro chick stick; nicotek metro midnight; nicquid; nicstick; nicstick dekang; nicvape; niin; nitro's cold brew; nixteria; njoy; no 7; no hype; no smok; no. 32 beard vape co.; nola bar; nomenon; noms; nube; nude; nude nicotine; nuport; nutricigs; nvee pro; obs; off the record; ohm boy; ohmboy; ok vape; okami; omari-o; omg; one hit wonder; one mad hit juice box; oooflavors; opmh project; orgnx; our daily bread; p.o.e.t. electronic nectar; p1; pachamama; paladin; palm; pancake man; patches by candy co.; pax; pegasus; pgvg labs; phillip rocke; phrut; phyre vapor; pink sticks; pinup vapors; ploom; ply rock; pod juice; pod mesh; pods; pop; pop clouds; pop vape; pop!; posh; posh vape; potters; premium ecigarettes; primitive vapor co.; primus vape co.; prism; progressigs; prohibition; propaganda; prophet premium blends; prosmoke; provape; provari; provog; psycho; puf cigs; puff; puff bar; puff labs; puff plus; puffmi; pure; pure cigs; pure extracts; pure smoke; purge; purilum; puro; pyro; qmos; quiet owl; quik; rad; randm; reds apple; regal cigs; rejuve; relx; revenge vapes; revive; revolution; rincoe; riot squad; rip tide ripstick; ripe; ripe vapes; riptide; river city vapes; rly gud; rokin; roll upz; rounds; rove; royal smoke; russian 91%; ruthless; ruyan jazz; rx vape; sadboy; salt bae; salt nic; salt nix; salteez; salty man; sapphyre nic; saveurvape; schwartz; sci-fi serum; sdmh; sea pods; select; seneca; sengoku; shenzhen fest technology co. ltd.; shft; shijin; sigelei; sigma; silverback; simple e-fruit; simply; simply eliquid; simply quit; sinister liquids; six realms; sixt; skwezed; skycig; slammin; smart smoke; smoant; smok; smok-e mountain; smoke free; smoke star; smokeless image; smoker's one choice; smokers 1 choice; smokestik; smoking everywhere; smokio; smpl; smy; snow wolf; snowplus; snowwolf; social cbd; solace; solaris; something; sonic vape; sorbae; south beach smoke; space jam; space vape; square; square 82; square drops; square e-head; square ehose; square gold; square reload; square t vape 2.0; squeezee; squid industries; steam engine; steamcrave; steem; steep; stig; stix; stlth; storz & bickel; strange clouds; strange fruit; strangers mods; strive; sugar lips; sugoi vapor; suicide bunny; suorin air; suorin air bar; superb; supherb; svoemesto; swell vape co.; swft; swisher; tab; taffy man; tailored vapors; targa; taste vapors; teardrip; tecc arc 5; tenx; teros; terpen; teslacigs; texas select vapor; the byrd; the candy man; the cuban; the custard shoppe; the drip factory; the mamasan; the milkman; the one; the standard 100; the standard vape; the vapor bar; the vapor chef; the vapor's knoll; timbr organics; tinted brew; titan; tobo; tony's; totally wicked; trailblazer; transistor; treats; trill classix; tsunami; tugboat; twelve monkeys; twirly pop; twisp; twist; twst; ud; uncle junk's; unique pods; urus; usa vape lab; utillian; uwell; v'nilla cookies & milk; v-gar 300; v2 cigs; v2 pro; vaal vapor; vale; vampire vape; vandy vape; vandyvape; vanvop; vapcigs; vape 100; vape canyon; vape craft; vape crusaders; vape dinner lady; vape heads; vapeco; vapefly; vapejoy; vapeleaf; vapelustion; vapen; vapengin; vapeonly; vaper; vaperev; vapergate; vapestick;

vapetasia; vapetek; vapin plus; vaping vamps; vapo; vapor 123; vapor cast store; vapor couture; vapor group; vapor maid; vapor shark; vapor storm; vapor x; vapor4life; vaporcade jupiter; vaporesso; vaporfi; vaporin; vaporlax; vapors anonymous; vaporx; vapour 50/50; vapour2; vapouriz; vapourlites; vaptasia; vaptex; vaptio; vapx; verdict; vertx; vertx plus; vessel; vethos design; vfeel; vgod; vibez; vicious ant; victory; vienti; vip; viper; vision; vivid; vivid vapours; vmr products; volcano ecigs; volish; von erl.; voodoo; voodoo & ego; voopoo; vq pods; vr labs; vrk; vuber; vuse; vx brandz; vyko; vype; wake mod co.; watson; westside vapor; wet; whip'd strawberry; whisl; white cloud; white cloud electronic cigarettes; wicked; wismec; wotofo; wotofo stentorian ram; wow vapor; wuuz; x hale o2; x hale o2 zero; x2c; xeo void; xfire; xhale vapor; xplorer; xtra; yami; yaya; yeti; yihi; yocan; yogi; yogi bar; yooz; yumapuff; z; z pods; zalt; zandera; zeltu; zenith; zeus; zig zag; zig-zag; ziip-lab; zonk e liquid; zooka; zoom; zoor; zoovoo; zuk vape

* We’ve noticed variations of several brands; although we list all variations here, some of them may be variations of the same brand: “bmor” vs. “b+mor”, “eversmoke” vs. “ever smoke”, “geekvape” vs. “geek vape”, “mpx” vs. “melting point extracts”, “mr. salt-e” vs. “mr. salt e”, “blu” vs. “myblu”, and “vandy vape” vs. “vandyvape”. We list all variations here because variations of the same brand may help users of this brand semantic database capture the corresponding unique brand mentioned in online stores and on social media.
